# Understanding variations in patient screening and recruitment in a multicentre pilot randomised controlled trial: a vignette-based study

**DOI:** 10.1186/s13063-016-1652-2

**Published:** 2016-10-26

**Authors:** Paul Hilton, Brian S. Buckley, Elaine McColl, Denise Howel, Douglas G. Tincello, Catherine Brennand, on behalf of the INVESTIGATE studies group

**Affiliations:** 1 Faculty of Medical Sciences, Medical School, Newcastle University, Newcastle upon Tyne, NE2 4HH UK; 2 Institute of Applied Health Sciences, University of Aberdeen, Aberdeen, Scotland UK; 3 Department of Surgery, University of the Philippines, Manila, Philippines; 4 Institute for Health and Society, Medical School, Newcastle University, Newcastle upon Tyne, NE2 4HH UK; 5 Department of Health Sciences, College of Medicine, Biological Sciences and Psychology, University of Leicester, Leicester, LE1 7RH UK; 6 Newcastle Clinical Trials Unit, Institute for Health and Society, Medical School, Newcastle University, Newcastle upon Tyne, NE2 4AE UK

**Keywords:** Randomised controlled trial, Equipoise, Screening, Recruitment, Eligibility, Vignette, Urodynamic investigation, Surgery, Stress urinary incontinence

## Abstract

**Background:**

The INVESTIGATE-I study was designed to inform a future definitive randomised trial of invasive urodynamic testing, compared to basic clinical assessment with noninvasive tests prior to surgical treatment, in women with stress urinary incontinence or stress-predominant mixed urinary incontinence. In a pilot randomised controlled trial, women from seven participating sites were screened, consented and randomised. Overall, 771 patients were identified from clinic notes and correspondence as being potential recruits and were sent the Patient Information Leaflet. Of those screened, 284 were deemed eligible, giving an overall ‘screen positive’ rate of 37 %. The numbers screened at individual centres varied between 14 and 399; the ‘screen positive’ rate varied between 22 and 79 % and the percentage of eligible women recruited varied between 55 and 100 %. The aim of this additional substudy was to explore why ‘screen positive’ rates may have varied so widely between apparently similar sites.

**Results:**

All 11 trial staff involved in screening in the seven recruiting sites were asked to evaluate a series of 20 identical vignettes, mainly based on actual general practitioner referral letters. Of the vignettes, 16 mentioned one or more definite inclusion criteria; the remainder had possible inclusions. Four had definite exclusions; 12 had possible exclusions. Free-text comments were sought to clarify the screeners’ decisions.

For six vignettes everyone agreed that the patient was eligible; for one all agreed she was not eligible; the breakdown for the remainder was mixed. Free-text comments illuminated uncertainties that may have led to variability in judging potential eligibility.

**Conclusions:**

Variability in judgements about potential trial eligibility highlights the importance of explicit and objective inclusion and exclusion criteria, and of agreed strategies for making judgements when information is missing. During the development and planning of trials, vignettes might be a valuable tool for training those involved in screening and recruiting patients, for identifying potential problems and ensuring greater consistency in the application of eligibility criteria.

**Trial registration:**

ISTCTN registry: ISRCTN71327395, registered on 7 June 2010.

**Electronic supplementary material:**

The online version of this article (doi:10.1186/s13063-016-1652-2) contains supplementary material, which is available to authorized users.

## Background

It is unclear why some trials reach recruitment targets more easily than others [1]. Some factors have been shown to be associated with more successful recruitment; for example, the research question itself (e.g. being a cancer or a drug trial), aspects of trial organisation (e.g. having a dedicated trial manager) and treatment access (e.g. involving a treatment that is only available within the trial) [2]. Other strategies, such as newsletters and mail shots, have been employed to encourage recruitment, although their effectiveness has not been established [2, 3].

The INVESTIGATE-I (INVasive Evaluation before Surgical Treatment of Incontinence Gives Added Therapeutic Effect?) study was a mixed-methods feasibility study, designed to inform the development of a definitive randomised trial of invasive urodynamic testing (IUT) (otherwise known as bladder function testing, or cystometry), compared to basic clinical assessment with noninvasive tests prior to surgical treatment, in women with stress urinary incontinence (SUI) or stress-predominant mixed urinary incontinence (MUI) [4]. A pragmatic, multicentre randomised pilot trial was conducted as part of the feasibility study to investigate how well units were able to identify eligible trial participants and recruit them. Initially, six full recruiting sites contributed to the pilot trial, with one further full site and two Patient Identification Centres (PICs) being incorporated later in an effort to improve recruitment. Site initiation and training visits were carried out by the chief investigator and the trial manager at each of the full recruiting sites.

**Fig. 1:**
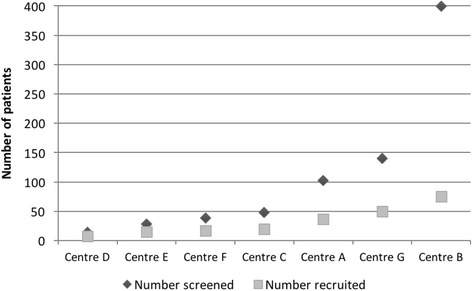
Numbers screened and recruited at individual centres. After Hilton et al. [4], reproduced under licence with permission of the authors

**Fig. 2:**
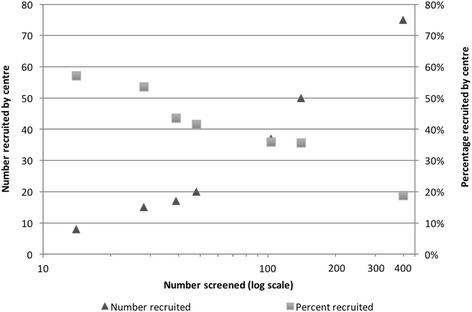
Number and percentage recruited to trial by number screened (shown on log scale) at each centre. From Hilton et al. [4], reproduced under licence with permission of the authors

At the trial sites a two-stage process was employed for identification and screening of potential participants. First, using predefined inclusion and exclusion criteria (see ‘Methods’), research nurses scrutinised clinic databases and identified potential recruits from hospital notes and correspondence. Second, those women identified as potentially eligible were sent Patient Information Leaflets (PILs) and invited to attend an in-person screening clinic, at which eligibility was confirmed. Overall, 771 patients were identified as being potential recruits, and were sent the PILs. Of those screened in person, 284 were deemed eligible for the trial, giving a ‘screen positive’ rate of 37 %. Despite the apparent consistency of screening methods, and the similar size of recruiting centres, the numbers reported on screening logs at individual centres varied between 14 and 399, with ‘screen positive’ rates between 22 and 79 %. The percentage of eligible women recruited varied between 55 and 100 %. Whilst the centres screening larger numbers of women also recruited larger numbers (see Fig. 1), the conversion from screening to recruitment decreased as the screening number increased (see Fig. 2).

In view of the variations seen in screening and recruitment, a quality assurance check was made with the principal investigators (PIs) and other recruiting staff in each unit. This confirmed that all employed similar procedures in relation to screening, in adherence with that laid out in the study protocol [5]. It was nonetheless possible that, although procedures were standardised and adhered to, variability in assessment of potential eligibility amongst recruiting staff may have resulted in variations in screening and recruitment.

Vignette-based studies have been used in many contexts over the last 50 years to aid understanding of phenomena across a wide range of scientific fields, including economics and marketing, as well as experimental, developmental and educational psychology [6]. Early vignette studies often used hypothetical scenarios for non-experimental purposes, for example in medical training and assessment [7]. Vignettes continue to be used for training purposes in several areas, notably professional ethics [8, 9], and have been particularly useful in research examining professionals’ attitudes, perceptions, beliefs [10] and decision-making [11]. Vignette-based studies have also been used to investigate both clinician enrolment behaviour [12] and patients’ willingness to participate in research [13, 14].

This paper reports an extension to the INVESTIGATE-I pilot trial aimed at exploring variations in patient screening and recruitment, using a vignette-based study of individual unit screeners.

## Methods

### Study design

A vignette-based study was conducted as an extension to a multicentre, pilot randomised controlled trial. Its aim was to explore consistency and variability of eligibility assessments made by those involved in screening hospital notes and communications to identify patients for recruitment.

### Participants and setting

All 11 trial staff (eight nurses and three doctors) involved in screening and recruitment in the seven full sites recruiting patients to the INVESTIGATE-I pilot randomised trial (PICs were not included).

### Materials and data collection

A set of 20 vignettes was developed that described fictional patients who might have been considered for the pilot trial. The vignettes were based wholly or largely on actual general practitioner referral letters and genuine patients, with identifiable information removed, and with some modifications to ensure that the whole range of inclusion and exclusion criteria were covered.

To be considered eligible for recruitment to the trial, women had to fulfil *all* the following criteria:

- Have a clinical diagnosis of SUI or stress predominant MUI
- Have stated that their family is complete
- Have undergone a course of supervised pelvic floor muscle training (PFMT) (with or without other nonsurgical treatments for their urge symptoms) with inadequate resolution of their symptoms
- Both the woman herself and her treating clinician should agree that surgery is an appropriate and acceptable next line of treatment

Additionally, any *one or more* of the following criteria excluded recruitment:

- Symptomatic pelvic organ prolapse (POP) requiring treatment
- Previous surgery for urinary incontinence (UI) or POP
- Urodynamic investigation within the last 3 years
- Neurological disease causing UI
- Current involvement in competing research studies, e.g. studies of investigation or treatment of UI
- Unable to give competent informed consent

Sixteen vignettes mentioned between one and three definite inclusion criteria (SUI [numbers 2, 3, 7, 8, 13, 14, 17 and 20], stress-predominant MUI [numbers 1, 4, 5, 6, 12, 15, 16 and 18], supervised PFMT [numbers 1, 4, 5, 6, 7, 8, 14 and 16] and family complete [numbers 7, 8, and 12]); the other four had possible inclusions (UI but not specified as to whether stress or urgency related [numbers 11 and 19], ‘wet all the time’ [number 9], PFMT mentioned but level of supervision not specified [number 10]).

Four had one or more definite exclusions (previous pelvic floor surgery [numbers 13 and 19]; neurological disease [number 15], did not wish surgery [number 13], urgency-predominant MUI [number 10]); and 12 others contained possible exclusions (PFMT not mentioned [numbers 2, 11, 12, 19] or not supervised [numbers 3, 9, 18 and 20], POP of uncertain significance [numbers 5 and 12], symptoms suggestive of overactive bladder (OAB) [numbers 17 and 18] or previous treatments for OAB [numbers 6, 9 and 16]).

Participants were circulated with an invitation to take part and a brief description of the proposed study (Additional file 1), the set of 20 vignettes (Additional file 2) and a score sheet (Additional file 3), were distributed through the web-based trial management site.

Participants were asked to assess the vignettes independently and indicate using the score sheet whether they would have considered the woman described in each to be potentially eligible for recruitment into the INVESTIGATE-I pilot trial. They were also asked to indicate whether their judgements were clear-cut or borderline, to provide information about the inclusion or exclusion criteria that informed their decisions and to make additional free-text comments as appropriate.

### Analysis

Each participant’s responses regarding whether each woman may be eligible were transformed into one of four possibilities in an ordinal scale: clear-cut ‘Yes’ (Y); borderline ‘Yes’ (?Y); borderline ‘No’ (?N); or clear-cut ‘No’ (N). All participants’ responses were summated to a majority grade within this four-point scale, and then rationalised to a majority ‘Y’ or ‘N’ grading. The majority decision was defined as one in which the percentage ‘Yes’ grading was above or below 50 %, irrespective of whether the decisions were considered to be clear-cut or borderline. An ‘expert’ protocol authors’ grading was also established for each vignette. Analysis was by descriptive statistics.

## Results

Each screener’s grading for the various vignettes is shown in Table 1. For six vignettes everyone agreed that the patient was eligible (percentage ‘Yes’ = 100 %); for one all agreed that the patient was not eligible (percentage ‘Yes’ = 0 %); the grade breakdown for the remainder was mixed, with percentage ‘Yes’ ranging from 9 to 91 %. The rates of ‘Yes’ or borderline ‘Yes’ judgements made by individual participants ranged from 45 to 80 %.

**Table 1:** Screener responses to the 20 vignettes

Vignette number	Centre (A–G) and Screener (1–2)																	
	A	B	C	D	B	A	E	F	G	C	F	%Yes	Grade breakdown				Majority/definitive grade	
	1	1	1	1	2	2	1	1	1	2	2		Y	Y?	N?	N		
8	Y	Y	Y	Y	Y	Y	Y	Y	Y	Y	Y	100 %	11	0	0	0	Y	Y
14	Y	Y	Y	Y	Y	Y	Y	Y	Y?	Y	Y	100 %	10	1	0	0	Y	Y
17	Y	Y	Y	Y?	Y	Y	Y?	Y	Y	Y	Y	100 %	9	2	0	0	Y	Y
4	Y	Y	Y?	Y?	Y?	Y	Y	Y	Y	Y	Y	100 %	8	3	0	0	Y	Y
7	Y	Y	Y	Y?	Y	Y?	Y?	Y	Y	Y?	Y?	100 %	6	5	0	0	Y	Y
1	Y?	Y	Y?	Y?	Y?	Y	Y?	Y?	Y?	Y	Y	100 %	4	7	0	0	Y?	Y
3	Y	Y	Y?	Y	Y	Y	Y	Y	Y	Y?	N?	91 %	8	2	1	0	Y	Y
20	Y	Y	Y	Y	Y	N	Y	Y	Y	Y	Y	91 %	10	0	0	1	Y	Y
6	Y?	Y?	Y?	Y?	Y?	Y	Y?	Y?	Y?	Y	N?	91 %	2	8	1	0	Y?	Y
12	Y?	Y?	Y	Y?	Y?	Y?	N	Y?	Y?	Y?	N	82 %	1	8	0	2	Y?	Y
16	Y	Y?	Y	Y?	Y?	N	N?	Y?	Y	N	Y	73 %	4	4	1	2	Y/Y?	Y
9	Y?	Y?	Y?	Y?	N?	Y	Y?	N?	N	Y?	Y?	73 %	1	7	2	1	Y?	Y
2	Y	Y?	Y?	Y?	Y	Y	N	N	Y	N	N?	64 %	4	3	1	3	Y	Y
11	Y?	Y?	N	Y?	Y?	Y	N	Y?	N	N	N	55 %	1	5	0	5	Y?/N	Y
5	Y?	N	Y?	Y?	N?	N?	Y	N	N	N	N?	36 %	1	3	3	4	N	N
18	N	Y?	N	N	N	N?	Y?	Y?	N	Y?	N	36 %	0	4	1	6	N	N
19	N	Y?	N	N?	Y?	Y	Y	N	N	N?	N	36 %	2	2	2	5	N	N
10	N?	N	Y?	N?	N?	N	N	N	N	N?	N?	9 %	0	1	5	5	N?/N	N
13	Y?	N	N	N?	N?	N?	N	N	N	N	N	9 %	0	1	3	7	N	N
15	N	N	N	N	N	N	N	N	N	N	N	0 %	0	0	0	11	N	N
% Yes (Y or Y?)	80 %	80 %	75 %	75 %	70 %	65 %	65 %	65 %	60 %	60 %	45 %							
‘Yes’ when majority ‘No’	2	2	2	1	1	1	3	1	0	1	0							
‘No’ when majority ‘Yes’	0	0	1	0	1	2	4	2	2	3	5							
Total ‘disagreements’	2	2	3	1	2	3	7	3	2	4	5							
Centre recruitment	37	75	20	8	75	37	15	17	50	20	17							

Data are sorted vertically by the rate of positive screening (% Y) for each vignette, and horizontally by % Y for individual screeners

*Y* = clear-cut ‘Yes’ decision; *Y*? = borderline ‘Yes’ decision; *N* = clear-cut ‘No’ decision; No? = borderline ‘No’ decision

For 14 out of the 20 vignettes, the majority decision was ‘Yes’. Although there were three vignettes for which there was disparity between the majority screeners’ grading and the experts’ grading in ‘clear-cut’ versus ‘borderline’ categorisation, there were no disparities in ‘Yes’ versus ‘No’ categorisation. This combined majority/expert ‘Yes/No’ categorisation was looked on as definitive and used in subsequent analysis.

There were 34 occasions on which one or more individual screeners ‘disagreed’ with the definitive categorisation. The number of ‘disagreements’ varied across the 11 screeners, ranging from 1–7/20 vignettes. Table 1 reports these separately as occasions on which the screener said ‘Yes’ when the definitive view was ‘No’, and those on which the screener said ‘No’ when the definitive view was ‘Yes’. The former judgement might be considered to be erring on the side of over-inclusiveness at the screening stage and was seen on 14 occasions; the latter judgement, erring on the side of over-exclusiveness, was seen on 20 occasions.

Ten of the eleven screeners made a total of 92 additional free-text comments to explain their judgements. The majority of comments related to missing information, most commonly whether PFMT had been undertaken at all, or whether it had been supervised. Although several screeners erred on the side of inclusiveness where such uncertainty existed (see Table 2), in some instances participants’ judgements about possible eligibility implied that they had taken the view that it had not been done rather than ‘might have been done’ (see Table 3). Other comments related to lack of clarity in the referral vignettes. For example, a number commented on reports of vaginal laxity or dragging sensation, although information about clinical findings in relation to POP was either missing or was negative (see Table 4). In some instances, participants’ judgements about possible eligibility implied that they took the view that these issues were the greater problem and the incontinence less of an issue. There were also uncertainties around the significance of descriptions of ‘rectal prolapse’ (as distinct from uterovaginal prolapse) and ‘repair surgery’ (not specifying prolapse repair). Finally, although individual participant’s judgements appeared to be largely consistent throughout the exercise, occasional aberrations were identified, seemingly inconsistent with their overall level of equipoise (see Table 5).

**Table 2:** Comments indicating inclusiveness in the approach to screening

Screener B1 (a high recruiting centre) excluded only 2 patients and commented on 5 vignettes with ‘I would send info’ e.g. ‘Even though physio isn’t mentioned, I would send info’ (patient 2); ‘Since no specific comment that woman requesting treatment for prolapse, I would send info’ (patient 3); ‘Although sounds urge-predominant, I would send info’ (patient 6); and ‘not enough in GP letter to exclude, so I would send info’ (patient 9).
Screener G1 (a high recruiting centre) excluded only 2 patients and commented on 7 vignettes with ‘I would try to contact patient prior to sending info’, e.g. ‘I would try to contact patient prior to sending info to check predominant symptom’ (patient 1); ‘I would try to contact patient prior to sending info to ensure no OAB symptoms’ (patient 2); ‘I would try to contact patient prior to sending info to check prolapse not significant’ (patient 7); and ‘I would try to contact patient prior to sending info to check has had physio’ (patient 12).

*OAB* overactive bladder

**Table 3:** Comments indicating exclusiveness in the approach to screening

Screener F2 (a low recruiting centre) excluded 11 women (5 of whom were included by the majority), on the grounds of missing information, e.g. ‘Need to check notes and if documented that patient has stress incontinence and received PFMT then would be eligible but if it is only on patient’s say so then further investigations would be beneficial to give a diagnosis’ (patient 3); ‘Would need to clarify what conservative measures patient had tried, if PFMT then would be eligible if family complete’ (patient 6); ‘No mention of PFMT and type of incontinence would need establishing and high possibility given age of having more children’ (patient 11).
Screener C2 (a low recruiting centre) excluded 8 women (3 of whom were included by the majority), e.g. ‘History suggests at least some OAB and has had urodynamics before’ (patient 16).

*OAB* overactive bladder, *PFMT* pelvic floor muscle training

**Table 4:** Comments suggesting lack of clarity in the vignette contributing to uncertain eligibility

Screener E1 (a low recruiting centre) excluded 7 women (4 of whom were included by the majority), e.g. they excluded one who reported ‘a dragging sensation’, but with no examination findings provided, on the grounds of ‘Patient has symptoms of prolapse’ (patient 12).

**Table 5:** Comment suggesting inconsistency in screener’s application of inclusion/exclusion criteria during the study

Screener G1 (a high recruiting centre) excluded only 2 women, both of whom were included by the majority, commenting ‘Would need urodynamics to determine the main symptom’ (patients 9 and 16).	

Differences between individual screeners and individual units were seen, although no clear patterns emerged. The rate of ‘No’ grading by screeners when the definitive grade was ‘Yes’ is perhaps the key to understanding recruitment. This statistic is mapped to centre recruitment (see shaded rows in Table 1), although again there was little evidence of relationship.

## Discussion

### Main findings

This vignette-based study revealed some interesting insights into challenges and opportunities affecting the screening of clinical notes and correspondence for potential trial recruits by multiple staff and centres. The majority ‘Yes’ versus ‘No’ grading of participants did not differ from the ‘definitive’ grading for any vignettes. However, complete consensus about potential eligibility was observed for only 7 of the 20 vignettes. Individual participants’ rates of positive judgements about potential eligibility varied widely, from 45 to 80 %. There were many occasions in which individual participants’ judgements differed from the definitive ‘Yes’ versus ‘No’ categorisation, with the number of disagreements ranging from 1 to 7/20 amongst participants.

Response data and participants’ explanatory comments about judging potential eligibility revealed tendencies towards both ‘inclusiveness’ and ‘exclusiveness’. Tending towards inclusiveness – judging a patient to be potentially eligible when there is a likelihood that they may be ineligible on subsequent, more detailed, in-person screening – may seem to offer potential for maximum recruitment. However, it was observed in the INVESTIGATE-I pilot trial that the more inclusive screeners were in their screening processes, the less ‘efficient’ they became on a case-by-case basis. As the number of patients invited for more detailed screening across sites increased, the number recruited also increased, but the proportion recruited (as a percentage of those screened in detail) decreased.

Both inclusiveness and exclusiveness - judging a patient to be potentially ineligible when they may be potentially eligible – may lead to potentially eligible patients not being invited to take part in a trial, and thus a theoretical potential for biasing the trial sample. Deciding upon a preferred approach in screening may ultimately be influenced by the resources available, and by the nature and burden of more detailed screening on research staff and patients. Limiting invitations for detailed screening to those patients who the screeners are more confident will be eligible may reduce both time spent in screening, and the proportion of patients subsequently found to be ineligible.

### Implications of findings

Variability amongst research sites and staff in judgements about potentially eligibility or differences in the application of inclusion and exclusion criteria are not the only factors that may result in difference in recruitment between sites, and no association was found in this study between participants’ tendencies towards inclusiveness and increased recruitment rates in their sites during the INVESTIGATE-I pilot trial. Other factors, such as differences in researchers’ experience, network of contacts, available time, and competing research pressures, may play a role. However, variability in the application of inclusion and exclusion criteria in the screening of trial participants has been observed in previous studies and has implications for the validity of a trial. A recent paper by Hubbard et al. reported that in a trial of cardiac rehabilitation in patients with bowel cancer admitted for surgery, the research nurse at one site screened a lower percentage of patients than the clinical nurse specialists on the other sites, having deliberately only assessed those she knew were most likely to be eligible [15]. A qualitative study of recruitment to the EaSTeR trial in early laryngeal cancer, found variability across surgeons in their application of inclusion and exclusion criteria, leading to variation between trial sites in rates of eligibility and decreasing the number of patients recruited [16]. Hamilton has suggested that this variability may have reflected differences in the degree of equipoise over the study intervention between PIs (Hamilton D, personal communication, 2016). Others have opined similarly, and have suggested that a distinction be made between personal or individual equipoise and community [4, 17] or academic equipoise [18, 19]. Given the consistency of screener responses within sites, it is possible that a similar effect may have been present in our study.

Weijer et al., in a survey of oncologists, used vignettes to explore whether subjective eligibility criteria led to greater investigator uncertainty and greater variability in decisions on patient inclusion [20]. Both hypotheses were supported, with the greatest differences being in respect of decisions that a patient was ineligible. Ideally, it would be best to avoid such uncertainties in the application of inclusion and exclusion criteria, so as to reduce the potential for over-inclusiveness and over-exclusiveness. To achieve this, it would be appropriate to ensure that definitions in inclusion and exclusion criteria are clarified and made as objective as possible and that strategies are developed to deal with initial screening data (i.e. in records or databases) that are missing or unclear. The strategies required will vary between trials, depending amongst other things, on specific study aims. One approach might be to develop decision-making algorithms for screeners based on key elements of missing information, so as to standardise when and how to seek additional information.

This study employed a vignette-based methodology to explore variations in screening and recruitment across a number of sites that had been involved in a previous trial. The methods used identified tendencies and uncertainties amongst the screening staff as a whole and at an individual level. Therefore, it seems likely that a similar approach could be used to advantage prospectively during trial development and planning. Donovan et al., in a qualitative interview study of the emotional consequences of equipoise on trial staff, found that training and support promoted greater confidence in equipoise and improved engagement and recruitment [17]. Vignette-based methods might be used in group training and standard-setting sessions for PIs and research nurses to identify and address areas of uncertainty in judging possible eligibility and to agree a consistent approach to the screening and recruitment processes across sites.

### Strengths and limitations

Although vignettes have been used in a number of research contexts, this is the first use of this methodology to explore variations in screening and recruitment of participants amongst research staff in a multicentre trial. Despite being a feasibility pilot trial, INVESTIGATE-I was one of the largest trials undertaken addressing this clinical question to date. All trial staff involved in screening in each of the study sites underwent similar instruction in screening methods during site initiation visits; all participated in the vignette study and returned a fully completed score sheet.

Proponents and critics of vignette research have raised concerns about the artificiality of vignettes [21, 22]. That is, textual descriptions and hypothetical behaviour might not be sufficiently representative of real-world phenomena, leading to concerns about the validity of research findings and conclusions based on them. The vignettes used in this study were wholly or largely based on actual general practitioner referral letters and genuine patients, and fulfilled relevant methodological recommendations [6].

## Conclusions

Variability in judgements over trial eligibility based upon hospital records and correspondence might be reduced by explicit and objective inclusion and exclusion criteria. Decision-making algorithms to assist screeners make judgements when information is missing may also improve consistency between centres. During the development and planning of trials, vignettes might be a valuable tool for training those involved in screening and recruiting patients, for identifying potential problems and ensuring greater consistency in the application of eligibility criteria. This method might be particularly applicable to the translation of feasibility studies into definitive trials.

## Additional files

Additional file 1: Letter of invitation to take part and instructions for screeners. (DOC 28 kb)
Additional file 2: Referral letters for evaluation of screening processes. (DOC 104 kb)
Additional file 3: Score sheet used in vignette-based study of screening processes. (DOC 86 kb)

## Abbreviations

INVESTIGATE: INVasive Evaluation before Surgical Treatment of Incontinence Gives Added Therapeutic Effect?
IUT: Invasive urodynamic testing
MUI: Mixed urinary incontinence
NIHR: National Institute for Health Research
OAB: Overactive bladder
PFMT: Pelvic floor muscle training
PI: Principal investigator
PIC: Patient Identification Centre
PIL: Patient Information Leaflet
POP: Pelvic organ prolapse
REC: Research Ethics Committee
SUI: Stress urinary incontinence
UI: Urinary incontinence
