# Supplementary material for: Understanding variations in patient screening and recruitment in a multicentre pilot randomised controlled trial: a vignette-based study
Source: Trials. 2016 Oct 26;17:522. doi: 10.1186/s13063-016-1652-2 (PMC5080689; doi:10.1186/s13063-016-1652-2)
Supplement: Additional file 1: — Letter of invitation to take part and instructions for screeners. (DOC 28 kb) [file 13063_2016_1652_MOESM1_ESM.doc]

## Additional file 1

### Letter of invitation to take part and instructions for screeners

Dear colleague,

We have had previous correspondence about the process of screening used in the INVESTIGATE-I study. The reason for this is that we had noticed quite marked differences between the various units not only in the numbers recruited, but also in the numbers they had to screen in order to achieve that recruitment. As far as we can tell, the hospitals themselves are broadly similar in workload etc., the patients are pretty much the same, and the description of what you did to identify patients for screening also seems to be much the same.

We are keen to investigate this further and propose to do this by a series of ‘dummy GP letters’ or vignettes for you and all others involved in screening in your unit to assess. There are 20 numbered vignettes in the attached file. Each consists of one or more communications from GPs, clinic notes, or Physiotherapy reports. Some are genuine letters, some made up; some are quite short, others more detailed. I hope this will not take up too much of your time, but in order to get a better understanding of this issue, a full return from all staff involved in screening of patients in all our study sites is quite important. Your replies will of course be kept anonymous, although it is important that we can identify the centre at which you work.

What we want to know is whether you would have considered each of the women described in the letters to be a potential recruit for the INVESTIGATE-I trial. In other words, if you had reviewed the letter at the time that we were looking for recruits into the trial would you, or would you not, have sent out a Patient Information Leaflet (PIL) to the woman described (*please* *tick either ‘Yes’ or ‘No’ in the blue boxes*). It would also be helpful to know whether you feel the decision is clear-cut, or borderline (*by ticking in the appropriate green box*), and something of why you made that decision (*by ticking the orange boxes and adding comments as appropriate*). A score sheet is provided as a separate attachment with this email; could you complete this for each patient and return to me by email at your earliest convenience? Many thanks and best wishes

Paul Hilton MD, FRCOG
Consultant Gynaecologist & Urogynaecologist
Royal Victoria Infirmary
Newcastle upon Tyne NE1 4LP
Tel: 0191-2825853; Fax: 0191-2825873
Email: paul.hilton@newcastle.ac.uk or [paul.hilton@nuth.nhs.uk](../paul.hilton@nuth.nhs.uk)
