# Supplementary material for: Understanding variations in patient screening and recruitment in a multicentre pilot randomised controlled trial: a vignette-based study
Source: Trials. 2016 Oct 26;17:522. doi: 10.1186/s13063-016-1652-2 (PMC5080689; doi:10.1186/s13063-016-1652-2)
Supplement: Additional file 2: — Referral letters for evaluation of screening processes. (DOC 104 kb) [file 13063_2016_1652_MOESM2_ESM.docx]

## Additional file 2

### referral letters for evaluation of screening processes

| Patient 1: **Mrs IS, D.o.B: 22.10.1963 Age: 48** |
| --- |
| This lady has been troubled by urinary incontinence for about 4 years. She has both stress and urge incontinence and sometimes wets herself without realising it. She has to wear a pad all the time even at night. She saw our trainee last year who referred her for physiotherapy. She says she has been doing the exercises as advised by the physiotherapists over the last 9 months but her symptoms remain the same. She has no children and other relevant past medical history. Her mum and sister both have problems with incontinence and her sister has had surgery for this. I would value your opinion and advice on management.  Yours sincerely |
|  |
| Patient 2: **Mrs KEW D.o.B: 07.05.1979 Age: 33** |
| Thanks for seeing this 33-year old lady who is suffering from stress incontinence. She suffers from incontinence on minor activity such as skipping, laughing and running. She is a keen athlete and the incontinence is particularly problematic when she is out running. She has had 2 normal deliveries which were uncomplicated. She did not experience incontinence after the deliveries, but describes her symptoms as having been bothersome in the last 2 years. She was told after the 2^nd^ delivery that she would be susceptible to prolapse but she has not had any sensation of anything coming down.  She has no current medication. Her only relevant past medical history is of abnormal smears and a loop biopsy of her cervix in March 2011.  Yours sincerely |

|  |
| --- |
| Patient 3: **Mrs MAA DoB: 23.06.1967 Age: 43** |
| This 43-year old woman is complaining of stress incontinence. She denies any urgency and says that when she coughs and laughs she passes small amounts of urine. She recently couldn’t take part in a charity run because of the incontinence. Mrs Atkinson had some frequency and nocturia x 1 to 2 but no dysuria or haematuria. She tells me that her sister had a similar problem and had a TVT. She has tried pelvic floor exercises including an internal pelvic toner to no avail. On examination the vagina appears normal; there is no demonstrable stress incontinence; and there was a small cystocele the uterus being well supported and the cervix healthy. She has a Mirena coil in situ. She had no other past history of note. I would very much appreciate your opinion regarding her future management.  Yours sincerely |
|  |
| Patient 4: **Mrs SH DoB: 11.05.1959 Age: 53** |
| This lady has mixed incontinence which is partially treated with Duloxetine. There is a large component of stress incontinence which interferes with her lifestyle. She is keen to explore any intervention you may be able to offer her.  Many thanks.  Yours sincerely  **Notes:**  The patient was seen in the outpatient clinic on the 22^nd^ May 2012 and subsequently referred for pelvic floor muscle training. Subsequent letter from physiotherapist as follows:  **Physiotherapy letter:**  Thanks for your letter referring Mrs H for outpatient physiotherapy for symptoms of stress incontinence. On examination she had good pelvic floor function except for anteriorly on the right which is deficient in bulk. Initially there was some improvement in symptoms but this has not been significant enough for her to feel confident and her leakage causes great embarrassment. I have discharged her from physiotherapy services and I would appreciate your review in clinic.  Yours sincerely  Clinical Specialist Physiotherapist in Women’s Health |

|  |
| --- |
| Patient 5: **Mrs JM DoB: 15.07.1976 Age: 36** |
| Thank you very much for seeing this lady who works as a Clinical Trials Manager in your hospital. She has been seen by the local Women’s Health Physiotherapists who complaints of mixed urinary continence symptoms and a sensation of vaginal laxity. With treatment her continence symptoms have improved but the sensation of vaginal laxity persists and she would like to discuss with you the possibility of surgical treatment. I enclose a copy of the physiotherapist’s assessment. Thanks very much for your help.  Yours sincerely  **Physiotherapy report:**  **Diagnosis:** Mixed continence symptoms  Sensation of vaginal laxity  **Outcome**  This lady has found benefit from conservative management to date. She is concerned however regarding laxity within the vaginal structures and would like referral to see a Consultant about this.  **History**  She was referred for physiotherapy on 14.2.2012. She has 3 children, 2 of which were delivered vaginally and one by caesarean section. She initially reported leakage with coughing or sneezing, and also some urgency. Physical examination revealed moderate pelvic floor muscle strain with moderate stamina and responsiveness. At the time of examination there was no significant vaginal or uterine vaginal or uterine descent.  **Management and outcome to date**  She has attended on 3 occasions in total and reports that her continence symptoms have become more manageable if not completely resolved. She is encouraged to undertake a programme of pelvic floor exercises but is keen to have referral to see a Consultant. |

|  |
| --- |
| Patient 6: **Mrs KEM D.o.B: 17.06.1979 Age: 33** |
| Thanks for seeing this 33-year old lady who is suffering from urinary incontinence for several years now. She has had two children by normal vaginal deliveries. She describes a mixed picture with both stress and urge symptoms. She also finds that she leaks during sexual intercourse. She has been seen by our nurses at our local incontinence clinic a couple of times, and for the last few years has been trying conservative measures.  Her symptoms are now severe and not improving. I have tried some oxybutynin whilst she waits for her appointment but in view of her severe symptoms I would appreciate your further assessment. She has a past history of pelvic inflammatory disease and irritable bowel syndrome; she currently has an IUCD in situ. |
|  |
| Patient 7: **Mrs ELA DoB: 20.04.1973 Age: 39** |
| Thank you for seeing this 39-year old lady who was previously under your care for the management of stress incontinence. At that time she preferred to undergo physiotherapy rather than surgery. Now however she is happy that her family is complete and physiotherapy has not improved her urinary symptoms. She has an additional bearing down sensation within the vagina but no external prolapse. I would appreciate your opinion as to surgical options for her ongoing symptoms.  Yours sincerely |
|  |
| Patient 8: **Mrs CA DoB: 24.02.1974 Age: 38** |
| Thank you for seeing this 38-year old lady who has been suffering from stress incontinence since the delivery of her 2^nd^ child 2 years ago. She has already seen a physiotherapist for pelvic floor exercises which she has been doing religiously. Her main problems are leakage of urine when laughing, coughing, sneezing and doing exercises classes. She does not seem to have any urge incontinence. She has to use incontinence pads every day. I have done a pelvic examination today which is normal. She has no sign of prolapse.  She is very distressed by her incontinence and would like to see you to discuss further treatment.  **Significant past problems**  Helicobacter eradication therapy April 2012  Gastroscopy abnormal March 2012  Anxiety states: October 2010  Asthma August 2000 |

| Patient 9: **Mrs EN DoB: 29.11.1950 Age: 62y** |
| --- |
| Thank you for seeing this lady who has had problems of urinary incontinence for over 30 years. She feels wet virtually all the time. She has been given advice leaflets about pelvic floor exercise, and a prescription for oxybutynin, but without improvement. She feels she can’t go on as she is, and is keen to pursue surgical options. Please see and do the needful. |
|  |
| Patient 10: **Mrs ZJ DoB: 11.6.1984 Age: 28y** |
| Thank you for seeing this young woman from Eastern Europe, with urinary incontinence for most of her life. She was troubled with bed-wetting as a child, and was under the care of the paediatricians and paediatric surgeons for many years. She underwent drug treatments, behavioural therapy, and some sort of open bladder operation at the age of 6 years (I’m afraid I don’t have details of this in our practice records). Her symptoms settled in her early teens, but since having her first child by a very traumatic forceps delivery, she reports increasingly troublesome urinary symptoms. Her bed is always dry, but she is up several times each night; she is wet on the slightest exertion, and says that she can never get to the loo on time. As a result, she needs to wear pads all the time, day and night. She has been trying to do pelvic floor exercises, but says she doesn’t feel anything much happening when she tries this. I have given her a trial of solifenacin, but again to little avail. She is keen to look at other treatment possibilities and I would be grateful if you could send her an appointment.  Her English is quite poor, and you would benefit from having an interpreter available.  Yours sincerely |

|  |
| --- |
| Patient 11: **Miss SR DoB: 24.9.1991 Age: 21y** |
| This young, single, mother of 2, is bothered by leaking urine. She attends Pilates classes but this if anything makes her leakage more bothersome. She is otherwise fit and well, and despite her age is very keen to consider surgery for this. I’d appreciate your assessment as to her appropriateness for this.  Yours sincerely, |
|  |
| Patient 12: **Mrs AMP D.o.B: 5.7.1980 Age: 32y** |
| Thanks for seeing this 32-year old lady who is suffering from urinary incontinence for several years now. She has had two children by normal vaginal deliveries. Her leakage is mainly related to exertion, and she also is troubled by a feeling of dragging in the vagina when lifting her children. She has a small cystocele on examination. Apart from a tubal tie 4 years ago she has no other history of note. I would welcome your opinion about the best approach to management.  Many thanks for seeing her  Yours sincerely, |
|  |
| Patient 13: **Mrs VW D.o.B: 23/11/45 Age: 67y** |
| I would welcome your reviewing this lady who complains primarily of stress incontinence. She was first seen about this over 30 years ago, and underwent a vaginal repair operation at ………… hospital then. She seemed completely free from leakage for about 10 years after that, but reports that her symptoms have been getting gradually more troublesome over the last few years. She is hypertensive, and suffers from occasional angina. She is currently prescribed lisinopril, bendroflumethiazide, and aspirin. She prefers to avoid further surgery if at all possible, but would welcome your opinion.  Many thanks for seeing her |

|  |
| --- |
| Patient 14: **Mrs DS D.o.B: 29/11/60 Age: 52y** |
| Thanks you for seeing Mrs S again. She has been bothered by stress incontinence for a number of years. She attended for assessment around 2004, and had some tests and physiotherapy then. This gave her some relief, although things never really settled completely, and she is keen to explore the possibility of more definitive treatment now.  Yours sincerely, |
|  |
| Patient 15: **Mrs JT D.o.B: 15/2/58 Age: 54y** |
| You may remember J, who has been known to have MS for the last 25 years; I think you looked after her in her last pregnancy in 1992. Her eyesight has been poor following an episode of optic neuritis about 5 years ago, although generally her neurological condition has been very stable, and her mobility is good. It is only in the last couple of years that she has been troubled by urinary symptoms, of which leakage is the most bothersome. She does occasionally struggle to get to the bathroom in time if she leaves things too long, but leakage on exertion has been the main concern for her. She feels she empties her bladder satisfactorily, and has not been bothered by urinary infection at all. Whilst she realises that the MS might be at the bottom of things, she would like to exclude other possible factors. She is under the care of Prof … in Neurology, and I’m sure she will be able to give you further details.  Many thanks for seeing her  Yours sincerely, |

|  |
| --- |
| Patient 16: **Mrs MJS D.o.B: 8/7/1975 Age: 37y** |
| Mrs S has recently joined our practice, having moved down from Scotland; we do not yet have access to her previous GP notes or correspondence. She has been treated in the past for urinary problems, and has had a number of medications, and says that she even had botox injections to her bladder. Since these latter interventions her symptoms have changed somewhat; previously she reported both urge and stress incontinence, but now she is left with only the stress element, with leakage occurring particularly on coughing or sneezing, or when she is at the gym. She has seen our local community continence advisor and tried pelvic floor exercises without any further improvement. She would appreciate an appointment to discuss her options.  She has no past history of note, and is taking no medications currently.  Many thanks for seeing her. |
|  |
| Patient 17: **Ms SJP D.o.B: 1/5/72 Age: 40y** |
| I would be grateful for you assessment of SJ. She has been bothered by stress incontinence for the last few years now. This initially occurred only on coughing and running, but now is a concern for her during sexual intercourse. There is no problem during manual stimulation, although with any sort of vaginal penetration she is worried that she will leak. Exercises have not helped, and she is keen to consider any alternative treatment approaches as this is having a significant impact on her current relationship.  Many thanks for seeing her. |

|  |
| --- |
| Patient 18: **Mrs JG D.o.B: 23/9/33 Age: 79y** |
| Mrs G came to see me for the first time last month. She complains of urinary incontinence on a daily basis, and this seems to be partly stress and partly urge related. We have tried her on oxybutynin in the last month, without benefit; we have given her advice about pelvic exercises, but have not been able to access specialist physiotherapy for her in the community. She has been a patient at our practice for the last came 40 years, but has attended on only 3 occasions in that time; I think this is a measure of just how much these symptoms bother her. I would welcome your opinion about further management.  She has no past history of note, and is taking no medications.  Many thanks for seeing her |
|  |
| Patient 19: **Mrs VD D.o.B: 31/4/54 Age: 58y** |
| I would appreciate your seeing this patient who is troubled by urinary incontinence. She has previously had surgery for a rectal prolapse, and now tends to constipation, but is otherwise in good health.  Yours sincerely |
|  |
| Patient 20: **Ms WD D.o.B: 15/6/57 Age: 55y** |
| This lady underwent hysterectomy for menorrhagia under care 3 years ago. She has been suffering from stress incontinence since that time. She says that she reported this to your trainee when she attended the review clinic at 6 weeks post-op. She was told this was probably just transient stress incontinence following the operation and was advised to do pelvic floor exercises. She has done these religiously over the last 3 years, but really feels things are not improving at all. She is wet on the slightest exertion, and requires to waer pads continuously day and night. I really feel she needs further investigation and treatment. I would be grateful if you could try to see her personally when she attends.  Yours sincerely |
