# Supplementary material for: Understanding variations in patient screening and recruitment in a multicentre pilot randomised controlled trial: a vignette-based study
Source: Trials. 2016 Oct 26;17:522. doi: 10.1186/s13063-016-1652-2 (PMC5080689; doi:10.1186/s13063-016-1652-2)
Supplement: Additional file 3: — Score sheet used in vignette-based study of screening processes. (DOC 86 kb) [file 13063_2016_1652_MOESM3_ESM.doc]

## Additional file 3

### Score sheet used in vignette-based study of screening processes
